# P-1476. Cefiderocol Treatment Outcomes and Risk Factors for Gram-negative Bacterial Infection Treatment Failure

**DOI:** 10.1093/ofid/ofae631.1646

**Published:** 2025-01-29

**Authors:** Keemia Rashidi, Christine J Kubin, Gary Wu, Brian Nelson, Anne-Catrin Uhlemann

**Affiliations:** NewYork-Presbyterian Hospital, New York City, New York; NewYork-Presbyterian Hospital, New York City, New York; NewYork-Presbyterian Hospital, New York City, New York; NewYork-Presbyterian Hospital, New York City, New York; Columbia University Irving Medical Center, New York, NY

## Abstract

**Background:**

The IDSA 2023 guidance lacks strong evidence about cefiderocol (FDC) efficacy and real-world data are limited. The aim of this study was to evaluate effectiveness, safety, and risk factors for clinical failure (ClinF) associated with FDC use in hospitalized patients.

**Methods:**

This was a retrospective, observational study. Adults treated with an initial course of FDC for ≥ 48 hours from Feb 2020 - Aug 2023 were included. Primary outcome was clinical success (ClinS) defined as a composite of survival, resolution of signs and symptoms, and absence of both recurrent infection or microbiological failure. Factors associated with success and failure were compared on univariable analysis and those with a p-value < 0.2 and FDC monotherapy were considered for inclusion in a multivariable model for ClinF.

**Results:**

87 patients were included. Most common pathogens: *Pseudomonas* spp (49%), *Acinetobacter* spp (29%), and *Klebsiella* spp 16%. Carbapenem-resistance was reported for 93% of isolates and majority of cases were from a respiratory source (72%). At FDC initiation, 39 patients (45%) were admitted to the ICU and the median PITT bacteremia score was 3 (IQR, 1.5-4). ClinS was achieved in 32 patients (37%). Within this composite, 70% survived, 59% resolved their signs/symptoms, 62% had absence of microbiologic failure, and 63% did not recur within 30 days. 16 patients (18%) developed FDC non-susceptibility (NS) within 90 days. ClinS was associated with urine and intra-abdominal sources, while ClinF was associated with COPD, mechanical ventilation, respiratory sources, and higher PITT bacteremia scores. No significant differences found between age, Charlson comorbidity index score, or monotherapy and combination therapy. COPD was found to be an independent factor associated with ClinF [OR 11.5 (95% CI 1.63-82.28, *p*=0.014)]. Comparing patients who developed FDC NS to those that did not, solid organ transplant [7(44) vs 14(20); *p*=0.088], longer antibiotic days prior to FDC [14.5 d vs 7 d; *p*=0.079], and longer FDC duration [14 d vs 10 d; *p*=0.023] were more common in those that developed NS.

**Conclusion:**

In this single center experience with mostly respiratory infections, FDC was successfully used with ClinF being associated with underlying illness. The potential development of NS should be further explored.

**Disclosures:**

**All Authors**: No reported disclosures

